# Rheumatoid Arthritis Veiled by Sickle Beta-Thalassemia: A Rare Immunological Association Delaying Diagnosis

**DOI:** 10.7759/cureus.17378

**Published:** 2021-08-23

**Authors:** Sahithi Sharma, Rakesh B M

**Affiliations:** 1 Cardiovascular Medicine, Mayo Clinic, Phoenix, USA; 2 Internal Medicine, Vydehi Institute of Medical Sciences and Research Centre, Bengaluru, IND

**Keywords:** sickle cell beta-thalassemia, rheumatoid arthritis, hashimoto’s thyroiditis, autoimmune diseases, haemoglobinopathies

## Abstract

Sickle beta-thalassemia is a rare variant of sickle cell disease (SCD) that manifests with milder symptoms. Musculoskeletal complications arising from this condition can mimic inflammatory arthritis and hence delay the diagnosis of rheumatoid arthritis (RA) until irreversible damage has been done. RA has been reported to occur with SCD but there is no documented literature thus far on its co-occurrence with sickle beta-thalassemia.

This case report elucidates the etiopathogenesis, clinical manifestations, and challenges encountered with the diagnosis and management of RA in a patient with sickle beta thalassemia.

## Introduction

India bears the second highest burden of sickle cell disease (SCD) in the world. A cross-sectional study conducted in Eastern India showed that the prevalence of hemoglobin disorders in the region was 12.17%, of which 0.26% were sickle beta thalassemia. There is a higher prevalence of hemoglobinopathies and rheumatological conditions in Eastern India when compared to the rest of the country [1]. 

Sickle beta-thalassemia (SBT) is a sickle cell variant syndrome characterized by the compound heterozygosity of sickle and beta-thalassemia genes. It is a combination of quantitative and structural abnormalities of hemoglobin, giving rise to a variety of clinical presentations. SBT is divided into sickle cell β+ thalassemia and sickle cell β0 thalassemia based on a decrease or complete absence of beta-globin synthesis, respectively. The symptoms in patients with sickle cell β+ thalassemia are less frequent and less severe than those in patients with homozygous SCD or sickle cell β0 thalassemia [2].

Musculoskeletal complications are common with hemoglobinopathies and range from vaso-occlusive crises with SCD to reduced bone density seen in beta-thalassemia. These manifestations may delay the diagnosis of inflammatory arthritis. Most often, irreversible joint damage and functional loss are already evident at the time of diagnosis of rheumatoid arthritis (RA) in these conditions [3]. In the case of persistent joint involvement, RA should be considered as a diagnosis, given the early and irreversible dysfunction related to the disease. SBT being a permanent inflammatory state due to vaso‐occlusive crises and hemolysis might increase the severity of RA [4]. For these reasons, early detection and intervention are critical to the progression and outcome of the disease. In this report, we describe a case of SBT with concurrent RA and Hashimoto’s thyroiditis, and explore the possible etiopathogenesis, outcomes, and challenges associated with the same.

## Case presentation

A 29-year-old woman hailing from West Bengal, India presented to the Department of General Medicine with pain and swelling in the small joints of both hands, wrists, and ankle joints associated with morning stiffness for eight months' duration. She was a diagnosed case of SBT on hydroxyurea and folate therapy with a history of recurrent transfusions. Diagnosis had been previously established by hemoglobin (Hb) electrophoresis. She also had Hashimoto’s thyroiditis leading to hypothyroidism, which was treated with thyroxine, 50 micrograms once daily.

On examination, the metacarpophalangeal and proximal interphalangeal (PIP) joints of both her hands were swollen and tender. There were no visible deformities. X-ray of the hand showed periarticular osteopenia. At the time of presentation, she had microcytic anemia with indirect hyperbilirubinemia, and elevated levels of erythrocyte sedimentation rate (ESR), C-reactive protein, thyroid-stimulating hormone, and anti-thyroid peroxidase antibodies. 

The differential diagnosis at the time was SBT-associated inflammatory arthritis vs RA. Infective and vaso-occlusive etiology was not considered due to location and lack of acute toxic symptoms. Persistent PIP joint involvement with chronic onset was more favorable for RA. Work-up revealed high positive rheumatoid factor (quantitative) and the subsequent qualitative anti-cyclic citrullinated peptide antibody test was positive; hence, the diagnosis of RA was confirmed.

The baseline investigations are included in Table 1.

**Table 1:** Baseline investigations WBC, white blood cells; RBC, red blood cells; PCV, packed cell volume; MCV, mean corpuscular volume; RDW, red cell distribution width; CRP, C-reactive protein; ESR, erythrocyte sedimentation rate; AST, aspartate transaminase; ALT, alanine transaminase; ALP,  alkaline phosphatase; RA, rheumatoid arthritis; CCP, cyclic citrullinated peptide; TSH, thyroid-stimulating hormone; TPO, thyroid peroxidase; Hb, hemoglobin.

Investigations		
Lab parameter	Observed value	Normal range
Complete hemogram		
WBC	10.4*10^9^/L	4.0-11.0*10^9^/L
RBC	2.43*10^9^/L	3.8-4.8*10^9^/L
Hemoglobin	8.6 g/dL	11.5-15.0 g/dL
PCV	26.80%	36.0-46.0%
MCV	99.30 fL	83.0-101.0 fL
RDW	10.40%	12.0-15.0%
Platelets	455*10^9^/L	150-410*10^9^/L
Acute phase reactants		
CRP	1.91 mg/L	<0.8 mg/L
ESR	84 mm/h	0-20 mm/h
Liver function test		
Total bilirubin	2.7 mg/dL	0.3-1.2 mg/dL
Direct bilirubin	0.14 mg/dL	0.0-0.2 mg/dL
Total protein	8.2 g/dL	6.4-8.3 g/dL
Albumin	4.0 g/dL	3.5-5.2 g/dL
AST	59 U/L	13-35 U/L
ALT	40 U/L	7.0-35.0 U/L
ALP	90 U/L	42-98 U/L
Sickling test	Positive	Negative
Rheumatoid testing		
RA factor	171 IU/mL	<20 IU/mL
Anti-CCP (qualitative)	Positive	Negative
Renal function test		
Urea	18.7 mg/dL	16-40 mg/dL
Creatinine	0.44 mg/dL	0.6-1.1 mg/dL
Sodium	134 mEq/L	136-145 mEq/L
Potassium	3.75 mEq/L	3.5-5.1 mEq/L
Calcium	8.9 mEq/L	8.6-10.0 mEq/L
Chloride	101 mEq/L	98-107 mEq/L
Urinary test		
Urea creatinine	61.4 mg/dL	16-327 mg/dL
Urea microprotein	19.0 mg/dL	1-14 mg/dL
Urea protein creatinine ratio	0.31	<0.2
Thyroid function test		
TSH	7.92 µU/mL	0.4-4.2 µU/mL
TPO antibodies	2.9 IU/mL	0-9 IU/mL
Hb electrophoresis		
HbA1	78.50%	95-98%
HbA2	3.10%	2-3%
HbF	2.30%	<0.5%
HbS	16.10%	0%

Chest X-ray, ECG, and two-dimensional echocardiogram revealed normal studies. 

ACR/EULAR 2010 classification score was determined as 8 (joints=3/5, serology=3/3, acute phase reactants=1/1, duration=1/1) indicating a definite clinical diagnosis of RA [5]. DAS 28 (disease activity score in 28 joints) was computed taking into consideration the number of swollen and tender joints, ESR, and global assessment of health [6]. The score was calculated as 5.8 out of 10, which implies high disease activity. 

She was initiated on hydroxychloroquine, 200 mg twice daily for RA after weighing the risk of chronic joint injury against the risk of infection. Blood counts, and liver and renal function tests monitored for adverse effects were within the expected range at the two-week follow-up. Further genetic testing was not carried out due to financial constraints of the patient. 

## Discussion

SBT includes a spectrum of diseases of increasing severity, which is determined by the heterogeneity of β-Thal mutations with concomitant HbS. This leads to different amounts of β-globin synthesis and consequently to different amounts of HbA. Higher levels of HbA are usually associated with a milder phenotype [2]. It is important to note that these cases could be wrongly interpreted as sickle cell trait since they have mild microcytic anemia [7]. SBT can present with musculoskeletal complications ranging from transient joint effusion to vaso-occlusive crises, osteomyelitis, septic arthritis, avascular necrosis of femoral head, early osteoporosis, and inflammatory arthritis.

Diagnosing RA in a patient with SBT can be challenging due to the similarity in presentation. RA usually affects small joints, as in this case; however, the musculoskeletal complications of SBT are likely to occur in large joints such as the knee. Inflammatory arthritis associated with SBT is usually acute, transient, and likely related to ischemia [8]. RA must be suspected when the symptoms are chronic, with the involvement of many small joints and the development of joint erosions/deformities. SBT-related arthropathy though usually not severe can worsen with concomitant steroid +/- methotrexate therapy. The overall mortality of patients in this group is 20% [9].

Literature connecting RA and SCD is available; however, we did not find any studies of SBT associated with RA. We postulate that the outcomes and manifestations with SBT will be similar to SCD, but less severe. RA in patients with SCD can further worsen the outcome and lead to irreversible joint injury and deformities. A study done by McFarlene et al. showed prolonged morning stiffness, increased periarticular osteopenia, and difficulty with activities of daily living among SCD-RA patients when compared to the control RA-only group [4]. Patients with SCD and autoimmune diseases have more frequent, severe SCD complications when compared to patients with SCD alone. They also have a higher incidence of chronic kidney disease, end-stage renal disease, avascular necrosis, pulmonary hypertension, and stroke [10].

Treatment of RA in patients with SBT includes the use of non-NSAIDs (non-steroidal anti-inflammatory drugs) for immediate pain relief, hydroxyurea for SBT, and early addition of disease-modifying antirheumatic drugs (DMARDs) to limit disease progression. Hydroxychloroquine was prescribed to our patient due to no known deleterious interactions with hydroxyurea [11]. Blood counts, and liver and renal functions were followed in our patient, which was normal at the two-week follow-up. Sulfasalazine, methotrexate, and anti-tumor-necrosis-factor alpha therapy have not been studied extensively in patients with RA and sickle cell hemoglobinopathies [11]. NSAIDs can worsen renal damage caused by SBT and steroid therapy is associated with increased risk of vaso-occlusive crises, hence complicating pain management in SBT. The presence of early osteoporosis deserves attention since SBT, RA, and steroids are independent risk factors for osteoporosis. 

The pathophysiology behind the increased incidence of autoimmune conditions with SBT can be explained by two mechanisms, with the first being defective activation of the alternate complement pathway in SCD, which predisposes to autoimmune diseases [12]. Alternatively, there are specific immunity genes near the β-globin locus at 11p15.5, which match with the autoimmunity susceptibility of the thalassemia trait. This suggests a strong and close genetic link at the same band changing the disease susceptibilities of thalassemia carriers [13]. However, coexisting autoimmune disease and SBT is rarely reported, suggesting possible underdiagnosis due to an overlapping of the symptoms. In this patient, there are two coexisting autoimmune conditions, namely RA and Hashimoto’s thyroiditis.

## Conclusions

Musculoskeletal complications of hemoglobinopathies can mask concurrent rheumatological conditions like RA. This can delay treatment until after irreversible damage has been done. The severity of RA in patients with SBT is higher than in those without it. Timely intervention can prevent disease progression. Management of RA in patients with SBT is complex due to the adverse effects of the commonly used medications on sickle cell hemoglobinopathy. Further studies are required to assess the role and safety of DMARDs in this class of patients. Genetic analysis to study the clustering of genes responsible for these autoimmune conditions (RA and Hashimoto’s thyroiditis) in patients with SBT may provide very interesting mechanistic insights into the pathobiology of autoimmune diseases.
